# Pharmaceutical Development of Nanostructured Vesicular Hydrogel Formulations of Rifampicin for Wound Healing

**DOI:** 10.3390/ijms232416207

**Published:** 2022-12-19

**Authors:** Chantal M. Wallenwein, Verena Weigel, Götz Hofhaus, Namrata Dhakal, Wolfgang Schatton, Svetlana Gelperina, Florian K. Groeber-Becker, Jennifer Dressman, Matthias G. Wacker

**Affiliations:** 1 Fraunhofer Institute for Translational Medicine and Pharmacology ITMP, Theodor-Stern-Kai 7, 60596 Frankfurt am Main, Germany; 2 Translational Center for Regenerative Therapies, Fraunhofer Institute for Silicate Research ISC, Neunerplatz 2, 97082 Würzburg, Germany; 3 Cryo Electron Microscopy, CellNetworks, BioQuant, Universitätsklinikum Heidelberg, 69120 Heidelberg, Germany; 4 Department of Pharmacy, Faculty of Science, National University of Singapore, 4 Science Drive 2, Singapore 117544, Singapore; 5 Klinipharm GmbH, Hauptstraße 23, 65760 Eschborn, Germany; 6 Faculty of Chemical and Pharmaceutical Technologies and Biomedical Drugs, D. Mendeleev University of Chemical Technology of Russia, Miusskaya pl. 9, 125047 Moscow, Russia

**Keywords:** hydrogel, marine sponge collagen, hyaluronic acid, rifampicin, liposomes, wound healing

## Abstract

Chronic wounds exhibit elevated levels of inflammatory cytokines, resulting in the release of proteolytic enzymes which delay wound-healing processes. In recent years, rifampicin has gained significant attention in the treatment of chronic wounds due to an interesting combination of antibacterial and anti-inflammatory effects. Unfortunately, rifampicin is sensitive to hydrolysis and oxidation. As a result, no topical drug product for wound-healing applications has been approved. To address this medical need two nanostructured hydrogel formulations of rifampicin were developed. The liposomal vesicles were embedded into hydroxypropyl methylcellulose (HPMC) gel or a combination of hyaluronic acid and marine collagen. To protect rifampicin from degradation in aqueous environments, a freeze-drying method was developed. Before freeze-drying, two well-defined hydrogel preparations were obtained. After freeze-drying, the visual appearance, chemical stability, residual moisture content, and redispersion time of both preparations were within acceptable limits. However, the morphological characterization revealed an increase in the vesicle size for collagen–hyaluronic acid hydrogel. This was confirmed by subsequent release studies. Interactions of marine collagen with phosphatidylcholine were held responsible for this effect. The HPMC hydrogel formulation remained stable over 6 months of storage. Moving forward, this product fulfills all criteria to be evaluated in preclinical and clinical studies.

## 1. Introduction

Most injuries to the human skin are followed by a well-coordinated healing process. However, a significant percentage of wounds are classified as chronic with a prevalence rate of 2% of the US population [1]. In these patients, the wound healing cascades are affected by underlying comorbidities such as obesity or diabetes. As a consequence, skin recovery is delayed significantly [2]. Considerable advantages of nanoformulations over conventional wound care products have been reported. They include controlled or sustained release of drugs, effective delivery of hydrophilic and hydrophobic agents, and improved cellular adhesion during the healing process [3,4]. For many years, rifampicin has been applied in the treatment of tuberculosis. However, more recent reports indicate potential applications in wound therapy due to a considerable anti-inflammatory effect [5,6,7,8]. Several in vitro studies investigated potential mechanisms. The inhibition of the lipopolysaccharide (LPS)-induced secretion of IL-1β and TNF, for instance, has been described in multiple cell lines including mononuclear cells, BV-2 microglial cells, RAW 264.7 macrophages, and primary microglia [5,6,7,8]. Patients suffering from non-healing wounds will likely benefit from topical rifampicin preparations that counteract inflammation and reduce the elevated production of proteolytic enzymes [9]. In animal studies, Sharma et al. and Gurel et al. both determined accelerated wound closure in the presence of rifampicin [10,11]. The main challenges in developing a topical delivery system of rifampicin are its poor aqueous solubility and rapid degradation in aqueous environments. The drug spontaneously degrades to rifampicin quinone (RQ) and 3-formyl rifamycin (3-FR) through oxidative and hydrolytic processes [5,12]. RQ represents the main degradation product, while hydrolysis to 3-FR is accelerated in an acidic environment [13,14]. While previous investigations were focused on the encapsulation of rifampicin into liposomes [15], the current study emphasizes the development of two nanostructured hydrogel formulations. They enable the topical administration of rifampicin, prolong the residence time of the drug, and enhance penetration into the wound bed. Liposomes were either embedded in hydroxypropyl methylcellulose or a combination of hyaluronic acid and collagen.

Cellulose derivatives are widely used in pharmaceutical formulations, especially for oral (tablets, capsules, and oral liquids) and topical (gels and ointments) administration [16]. They are suitable gelling agents due to their biocompatibility, low cost, and hydrophilic properties [17]. Therefore, as a first step, several cellulose derivatives were evaluated to embed the liposomes into the gel matrix.

In a second approach, we used hyaluronic acid and marine collagen extracted from *Chondrosia reniformis* Nardo. This sponge is collected from the Mediterranean Sea and exhibits a lower risk of viral cross-species infection than collagens extracted from other sources [18]. Although there are several potential contaminants such as heavy metals or microplastics, the excipient can be considered safe when administered topically due to the low risk of systemic exposure [19,20]. More than 20 types of collagen have been identified, and some of them are being used in drug development [21]. In this context, beneficial pharmacological effects in wound healing play an important role. A reduction in the levels of matrix metalloproteases and elastases in chronic wounds and, thus, an improvement in the formation of granulation tissue were reported [22,23]. Primarily, these biomaterials are extracted from animal tissue, but a rising number of studies consider marine collagen as a safer alternative [18]. Hyaluronic acid is a major component of the extracellular matrix. It promotes wound healing by accelerating tissue repair and, thus, the formation of granulation tissue [24]. The viscoelastic gels absorb a considerable amount of water and improve the hydration of tissues [25,26].

After the development of these two nanostructured hydrogel formulations, a freeze-drying method was established. The process resulted in well-defined lyophilized cakes characterized by flawless visual appearance, rapid redispersion times, and low levels of residual moisture. However, the morphological characterization of the liposomes embedded in the hyaluronic acid–collagen gel revealed an increase in particle size that was confirmed by subsequent release studies. An interaction between collagen and phosphatidylcholine was elucidated by investigation of the morphology in the dried state.

## 2. Results and Discussion

### 2.1. Gel Matrix Based on Hydroxypropylmethylcellulose

Cellulose derivatives were selected as gelling agents for the hydrogel formulation due to their solid track record in clinical dermatology, biocompatibility, low cost, and hydrophilic properties [17].

Initially, two different concentrations of hydroxypropyl methylcellulose (HPMC, 2.5% and 3%), methylcellulose (MC, 2% and 2.5%), and hydroxyethyl cellulose (HEC, 2% and 2.5%) were added to the dispersions comprising 5% sucrose for the subsequent freeze-drying process (Section 3.2.1). The resulting freeze-dried formulations were broadly distributed in size with a polydispersity index (PDI) > 0.4, and several intensity peaks in a range from 100–200 nm were revealed by DLS. Most likely, the drying process of the partially melted product led to interactions between the liposomes followed by their coalescence into larger vesicles.

**Figure 1:**
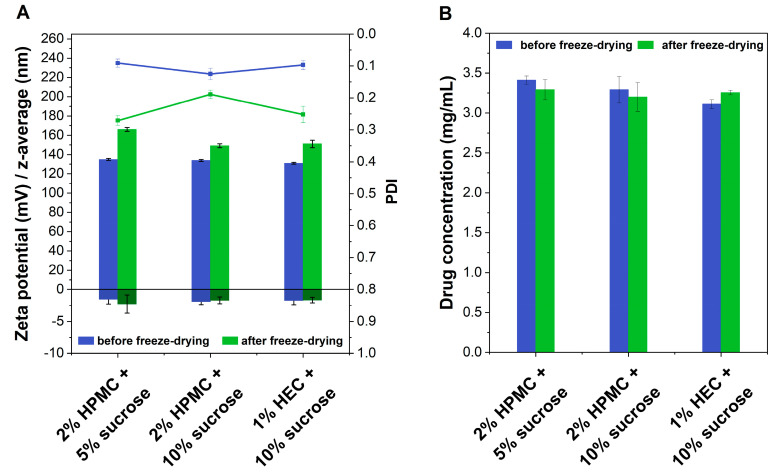
Comparison of three different formulation approaches using various celluloses before and after freeze-drying with (**A**) presenting the physicochemical characteristics including particle size (left axis, light green and blue bars), zeta potential (left axis, dark green and blue bars), and PDI (green and blue line) and (**B**) indicating the drug content (Mean ± SD, n = 3).

Additionally, the samples comprising MC could not be reconstituted; thus, this gelling agent was not used in the subsequent experiments. Accordingly, the composition of the gels was altered. Lower concentrations of HPMC (1% and 2%) and HEC (0.5% and 1%) were added to the liposomes comprising two different sucrose concentrations of 5% and 10%, respectively (Section 3.2.1). The three most promising formulation prototypes were identified (Figure 1). Before freeze-drying, all formulations exhibited a vesicle size distribution corresponding to the rifampicin liposomes with a PDI < 0.15 and a mean particle diameter of approximately 130 nm. The surface charge was slightly negative, which is typical for the phospholipids employed during liposome preparation. After freeze-drying, the mean particle size and PDI increased for most formulations. The zeta potential did not change much for all three formulations. Furthermore, all three formulations exhibited a redispersion time below 2 min. The formulation comprising 2% of HPMC and 10% of sucrose exhibited considerable advantages due to a PDI of <0.2 compared to the other two formulations. Regarding the drug content, all three formulation prototypes remained stable (Figure 1B).

**Figure 2:**
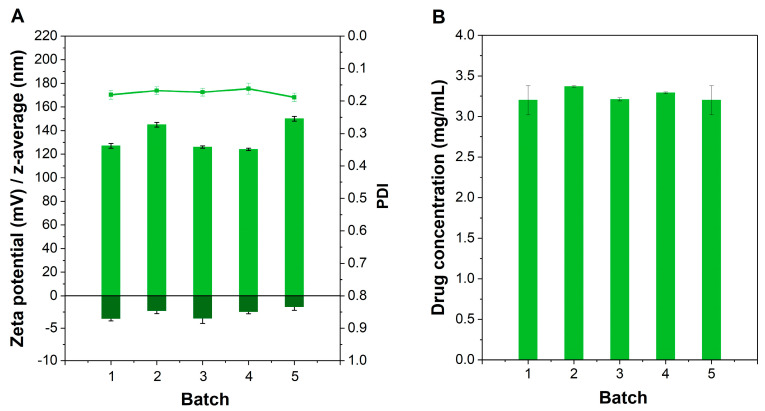
Batch reproducibility of HPMC gel manufacture with (**A**) indicating the reproducibility of five batches regarding their physicochemical characteristics including particle size (left axis, light green bars), zeta potential (left axis, dark green bars), and PDI (green line) and (**B**) presenting the drug content of different batches (light green bars) after freeze-drying (Mean ± SD, n = 5).

Hence, this formulation (2% HPMC, 10% sucrose) was selected for further investigation. The reproducibility of the manufacturing process regarding the physical stability and drug content is presented in Figure 2.

The residual water content of 0.70% ± 0.02% of these lyophilizates was determined using volumetric Karl Fischer titration as described in Section 3.2.1. This value indicated good storage stability and is evaluated in more detail in Section 2.4.

### 2.2. Gel Matrix Composed of Hyaluronic Acid and Collagen

**Figure 3:**
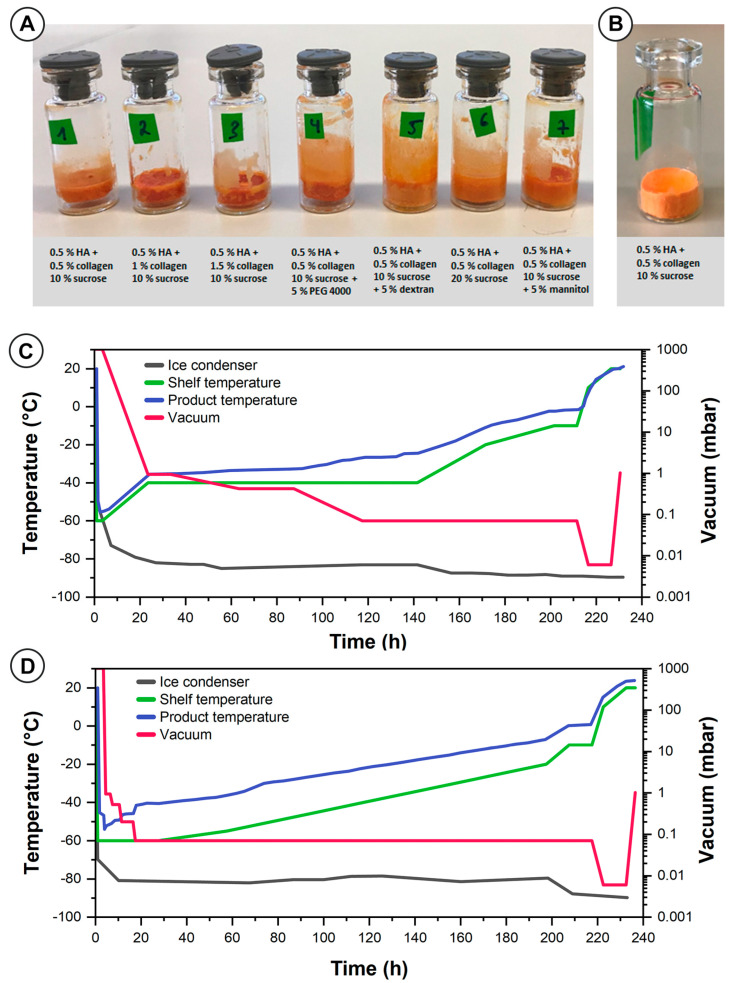
Representative examples of the freeze-dried collagen–hyaluronic acid hydrogels with rifampicin-loaded liposomes before (**A**) and after (**B**) process optimization together with the process records. After a first process optimization, the configuration (**C**) did not lead to a well-defined product irrespective of the addition of cryoprotectants and lyoprotectants. After further process optimization, a well-defined cake (**B**) was achieved. The final process parameters are also presented (**D**).

To support the therapeutic effects of rifampicin, the second hydrogel matrix was developed using collagen extracted from *Chondrosia reniformis* Nardo [27] and hyaluronic acid. Initially, we used the freeze-drying process developed for hydrogels composed of the semisynthetic cellulose derivative (Table S1 and Table 1A). This freeze-drying process resulted in collapsed cakes of all hydrogel formulations after drying (Table 1A). Marine collagen requires significant swelling time and, accordingly, the water content and matrix density represent a major challenge for further processing. As a result, the following parameters of the freeze-drying process were adjusted as presented in Figure 3C,D: the shelf temperature, the vacuum, and the drying time during primary drying. The optimizations included that the primary drying was carried out at a slightly increased vacuum of 0.07 mbar, and, with an additional step at 0.42 mbar, the vacuum was lowered more gently from 0.94 mbar to 0.07 mbar. Furthermore, the shelf temperature was initially raised from −60 °C to −40 °C instead of −30 °C during the primary drying step, and the total primary drying time was increased by 152 h (Figure 3C). Even after this adjustment of the freeze-drying process and the addition of a wide variety of lyoprotectants and cryoprotectants, as well as a reduction of the product load per vial, the material melted during the secondary drying without resulting in an acceptable lyophilizate (Figure 3A, Table 1B). In addition, some of the lyophilizates required more than 4, (Table 1(B5,B7), 8 (Table 1(B6)), or 10 min (Table 1(B1,B2,B4)) for redispersion.

Freeze–thaw studies were used to identify suitable cryoprotectants. Ozcelikkale et al. reported a destabilizing effect of freezing on collagen fibrils due to the considerable growth of ice crystals [28]. DMSO and PEG 400 were identified as potential stabilizers but did not provide any advantage in the subsequent freeze-drying process (Table 1C).

After further process optimization, we increased the number of intermediate steps to reduce the vacuum from 0.94 mbar to 0.07 mbar, along with a gradual increase in the shelf temperature by 5 °C every 20 h from −55 °C to −20 °C; once a vacuum of 0.07 mbar was reached, the parameter set led to a well-defined freeze-dried cake (Figure 3B,D). Additional quality parameters included the redispersion time, the residual water content, and characterization of the embedded liposomes after redispersion and separation of vesicles from the gel matrix. The formulations comprising 0.5% of HA and 0.5% collagen supplemented with either 10% sucrose (Table 1(C1)), 10% sucrose, and 5% PEG 4000 (Table 1(C4)), or 10% sucrose and 5% dextran (Table 1(C5)) exhibited an acceptable redispersion time of below 2 min. With 2.11% ± 0.18%, the residual water content of the lyophilizates was higher compared to the cellulose hydrogels. This could be due to the high water-binding capacity of HA and collagen, together with a complex three-dimensional structure potentially providing small cavities with water strongly bound to the surface. Nevertheless, this water content remained within acceptable limits [29].

Quantifying the particle size of the liposomes after redispersion of the hydrogel was more of a challenge. DLS measurements are based on the intensity fluctuations of light scattered at the particle surface [30,31,32]. Over time, particularly during the drying process, the drying and swelling may lead to increased background scatter due to incomplete separation of the vesicles from the matrix material. Accordingly, the particle size determined after freeze-drying was broadly distributed in size (Table 2). To better understand the morphological changes of the vesicles, we further analyzed the cryo-TEM micrographs.

### 2.3. Characterization of the Morphology by Cryo-TEM and TEM

**Figure 4:**
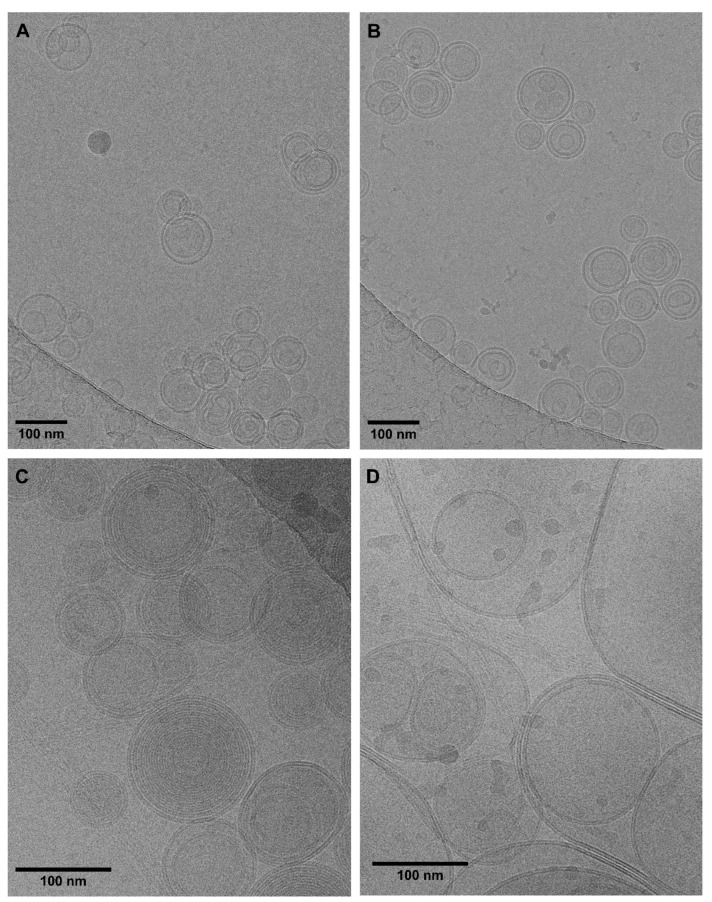
Visualization of the vesicles embedded in the HPMC gel before freeze-drying (**A**) and after redispersion of the freeze-dried product (**B**) using Cryo-TEM. The micrographs of the liposomal collagen–hyaluronic acid gel before freeze-drying and after redispersion of the lyophilized cake are presented in (**C**,**D**), respectively.

Electron microscopy was used to investigate the morphological characteristics of both the rifampicin-loaded liposomes and the gel structure. Cryo-TEM is most suitable for the characterization of liposomes in the dispersed state [32,33]. Representative micrographs of the liposomal HPMC gel before freeze-drying and after redispersion of the lyophilized cake are presented in Figure 4A,B. The mean particle size of approximately 100 nm determined by DLS is confirmed by these images. Mostly multilamellar and only a few unilamellar vesicles were observed. Generally, the freeze-drying process had little impact on the size and morphology of the liposomes.

For the collagen–hyaluronic acid hydrogel, the observed diameter of approximately 100 nm (Figure 4C) before freeze-drying corresponded to the outcomes obtained by DLS. The liposomes exhibited multilamellar structures similar to those embedded into the HPMC gel. After redispersion, the particle size of the liposomes embedded into the collagen–hyaluronic acid hydrogel could not be determined reliably using DLS. We assumed that changes in the density characteristics of marine collagen led to the incomplete separation of the liposomes from the matrix. This was supported by a decrease in the zeta potential after freeze-drying. Therefore, we additionally determined the size distribution from the cryo-TEM micrographs. Martin’s diameter of 100 vesicles was measured, confirming an increase in the vesicle size from 113 ± 1 to 410 ± 134 nm. For the redispersed formulation, larger vesicles with reduced lamellarity and a wide variety of shapes and sizes were observed (Figure 4D). Moreover, the characteristic fibril structure of collagen was observed.

While the morphological characteristics of the vesicles are not affected by HPMC, the combination of hyaluronic acid and marine collagen altered the properties of the formulation significantly. Hence, we used conventional TEM to observe these characteristics in the dried state. Although several steps of staining and dilution are required, this potentially provides deeper insight into the changes induced by the freeze-drying process.

**Figure 5:**
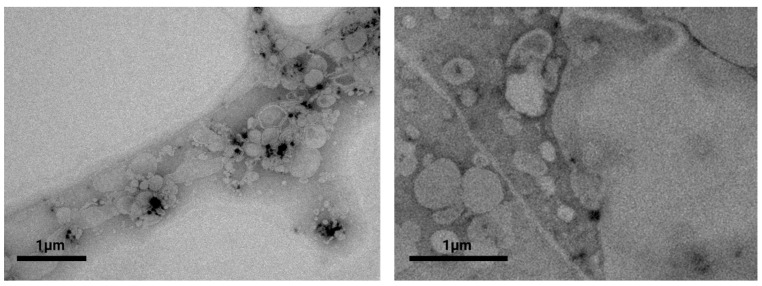
TEM micrographs of the collagen–hyaluronic acid hydrogel obtained in the dried state show a 4800-fold (**left**) and 6800-fold magnification (**right**).

Under these experimental conditions, all constituents of the formulation contribute to the morphology including collagen, hyaluronic acid, the rifampicin-loaded vesicles, and sucrose. The solid fraction was arranged along the collagen fibrils forming particle structures of various sizes. Vesicles in the expected size range still occurred; however, the collagen network affected the morphology considerably (see Figure 5). We believe that the increase in size was mostly due to the interactions between collagen and phospholipids. The material acted as an adsorber and absorber during the drying process. Despite being responsible for a considerable increase in the particle size, the resulting nanogel still provided an interesting three-dimensional structure that deserved further investigation.

### 2.4. Investigation of the Stability

**Figure 6:**
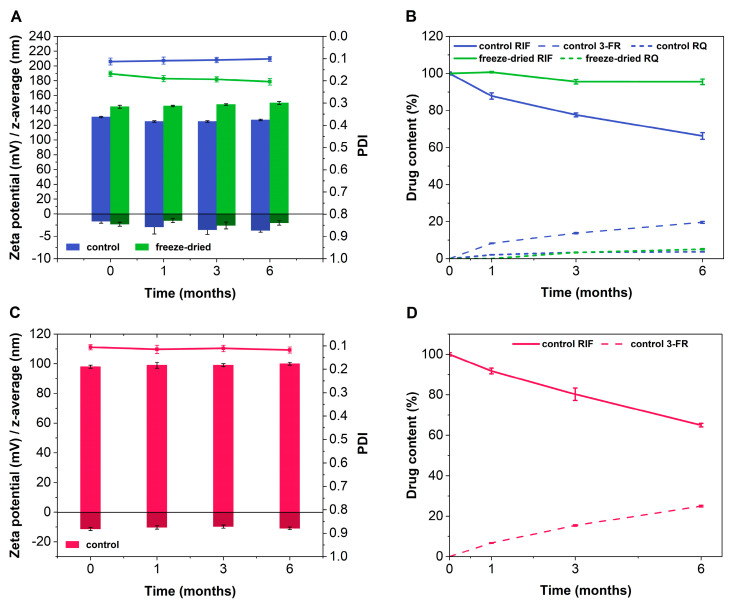
Comparison of physical (**A**,**C**) stability including particle size (left axis, light green, blue and red bars), zeta potential (left axis, dark green, blue and red bars), and PDI (green, blue, and red line) and chemical (**B**,**D**) stability of HPMC gel (**A**,**B**) and hyaluronic acid plus collagen gel (**C**,**D**) over 6 months (mean values ± SD, n = 3).

Our findings indicated a minor influence of cellulose on liposomes. Hence, the physical stability of the HPMC hydrogel before and after freeze-drying was investigated over 6 months when stored in a refrigerator (5 °C ± 3 °C). The physical stability of the dispersion (Figure 6A, blue) and lyophilized product (Figure 6A, green) was almost identical. However, the chemical stability was significantly improved by lyophilization. In the presence of water, rifampicin may undergo hydrolysis to 3-FR or oxidize to RQ. The rifampicin content of the aqueous dispersion decreased over time, while an increasing amount of 3-FR was determined. This indicates hydrolysis of the drug molecule. For both formulations, approximately 5% of the drug was oxidized to RQ. Overall, after 6 months of storage, approximately 95% of the initial rifampicin concentration was found in the freeze-dried formulation (Figure 6A,B), while only 66% was maintained for the dispersion.

Furthermore, we examined the physical stability of the collagen–hyaluronic acid gel. For this investigation, we did not include the freeze-dried product due to the limitations of routine analytics to accurately determine the particle size. When investigating the stability of the dispersion, the liposomes remained stable over 6 months (Figure 6C). However, a 35% decrease in the drug content was observed (Figure 6D).

A reduction in the drug content disqualified the aqueous dispersion comprising collagen and hyaluronic acid for further investigations. Hence, we decided to study the behavior of both freeze-dried formulations in more detail. The morphological differences could potentially lead to very different characteristics that could be of interest in wound-healing applications.

### 2.5. In Vitro Drug Release

**Figure 7:**
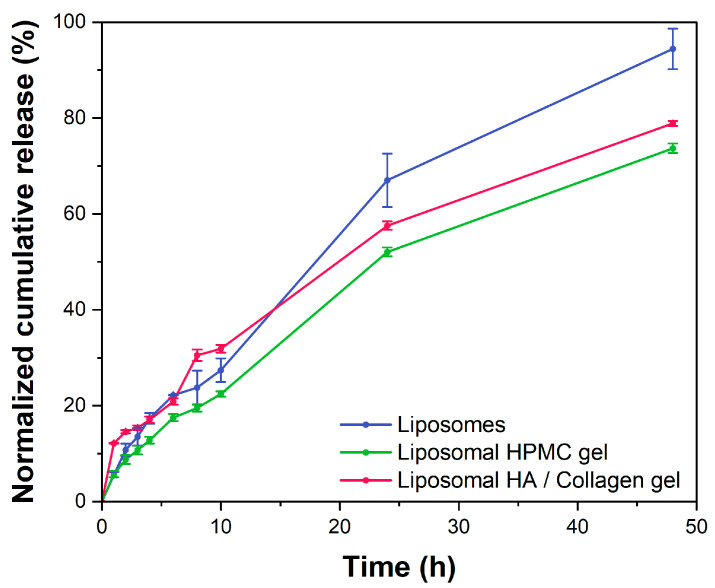
Comparative release study with the two nanostructured hydrogels and the liposomal dispersion (mean ± SD, n = 3).

The release of rifampicin from the two freeze-dried liposomal hydrogels and the liposomal dispersion was investigated using dispersion releaser (DR) technology [30,34,35]. After determining the membrane permeation coefficient of rifampicin (2.24 × 10^−3^ ± 0.09 × 10^−3^ cm^2^∙h^−1^), the release profiles were normalized to account for the influence of the analytical method on the release profile [34,36,37]. The DR provides a non-physiological environment that accelerates the release and membrane permeation. Moreover, the method subjects the formulation to constant shear. Under these conditions, the release behavior is more affected by the affinity of the drug molecule to the excipients than by the formation of a three-dimensional gel network. Hence, the integrity of the vesicle system can be measured. A liposomal dispersion of rifampicin was used as the control (Figure 7, blue line). Initially, the release rate of the HPMC gel was slightly lower compared to the liposomes (Figure 7, green line). Even at this shear rate, a certain influence of the increased viscosity on the release was to be expected. On the contrary, the collagen–hyaluronic acid gel exhibited a slightly but not significantly higher release rate during the first hour. This indicated a reduced encapsulation of rifampicin (Figure 7, red line) and became a statistically significant difference at 8 and 10 h (ANOVA, *p* < 0.05).

After 48 h, approximately 94% of rifampicin was released from the liposomal dispersion in contrast to 74% from the HPMC gel and 79% from the collagen–hyaluronic acid gel. The release of rifampicin from the collagen–hyaluronic acid network during the first 10 h strongly indicated a reduced encapsulation of rifampicin after freeze-drying. Hence, the collagen potentially interacted with the phospholipids to an extent that the drug binding capacity was reduced significantly.

Taken together, the observations made by electron microscopy were confirmed by the release study. Albeit a pharmacologically interesting excipient, the freeze-drying process led to an unstable dispersion of rifampicin. Hence, a liposome gel based on this material did not lead to optimal stability characteristics as required for the development of a topical drug product. Considering the tremendous effort of the freeze-drying experiments, other materials than the phospholipids (with lower affinity to the slow-swelling collagen network) may be more suitable for the formulation of rifampicin, collagen, and hyaluronic acid. Albeit stable in the liquid state, the shelf-life remains critical to the performance of the formulation. Therefore, this formulation approach was discontinued, and further studies were focused on the characterization of the HPMC hydrogel.

### 2.6. Rheological Characterization of the Nanostructured HPMC Gel

The rheological behavior and viscosity of dermal formulations not only affect spreadability and skin feel but also the residence time of the drug at the administration site [38,39].

**Figure 8:**
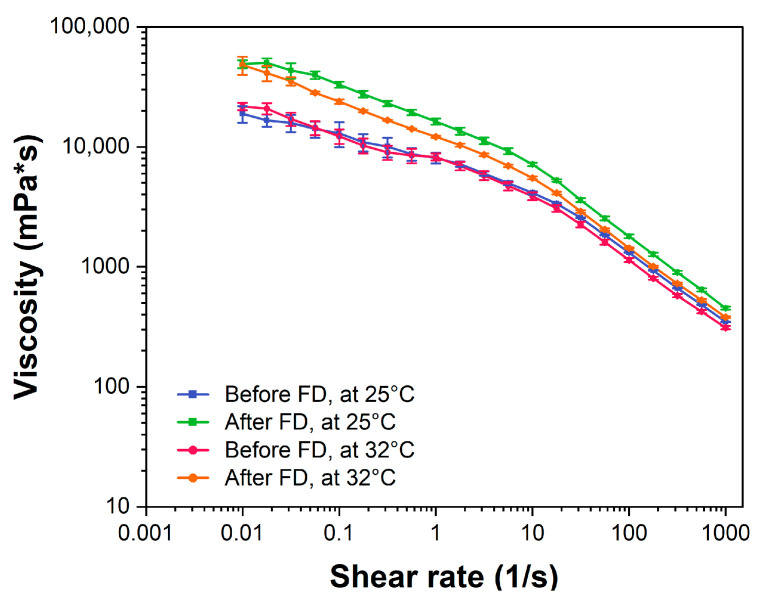
Comparison of the viscosity of the nanostructured HPMC gel before and after freeze-drying at two different temperatures (25 °C and 32 °C) (mean ± SD, n = 3).

To investigate the impact of the colloidal dispersion on the rheological behavior of the formulation, the characterization was performed using a 2% HPMC hydrogel at 25 °C as a reference. A viscosity of 4255 mPa·s was found. HPMC hydrogels exhibit pseudoplastic behavior (shear thinning) as confirmed by our measurements and the previous literature [40]. To determine the flow behavior of the nanostructured HPMC gels after manufacturing and after freeze-drying, the dynamic viscosity was measured between shear rates of 0.01 and 1000 s^−1^ at 25 °C and 32 °C as described in Section 3.9. For all gel formulations viscosity decreased with increasing shear rates (see Figure 7), indicating a shear-thinning (pseudoplastic) flow behavior as determined for the pure HPMC gel. Thus, the liposomal carrier did not affect the flow behavior but resulted in a considerable increase in viscosity (4–5-fold) probably due to the high amount of lipid used (80 mg/mL) and the addition of 10% sucrose. An increase in temperature had only little influence on the viscosity. A difference in viscosity was obtained for the formulations measured after manufacturing (Figure 8, blue line and red line) and the redispersed formulations after freeze-drying (Figure 8, orange and green line). After freeze-drying, a twofold increase in viscosity was determined. Possible reasons could be the competition of the gelling agent and lipids for the water during redispersion and shorter swelling times of the HPMC as the samples were directly reconstituted using a vortex after the addition of ultrapure water.

In conclusion, the shear-thinning properties of the formulations are beneficial for the application of dermal preparations on the skin as they facilitate manual application resulting in higher patient compliance.

### 2.7. In Vitro Skin Irritation of Rifampicin Liposomes and HPMC Gel

**Figure 9:**
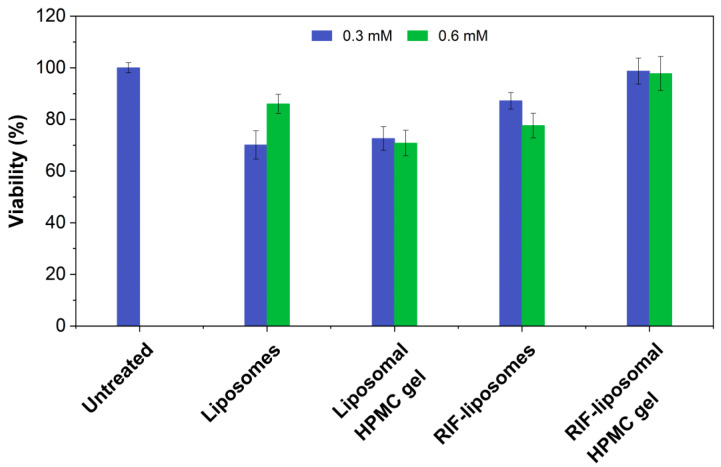
Investigation of the in vitro skin irritation of the rifampicin-loaded liposomes and the nanostructured HPMC gel with rifampicin in comparison to a rifampicin solution. As a control, the vehicles without rifampicin were also examined.

The percentage of viable cells after exposure to the various preparations is presented in Figure 9. Values below or equal to 50% indicate a potentially irritating effect of the preparation [41]. Overall, neither the liposomal preparation nor the hydrogel led to a considerable decrease in cell viability. This includes both the preparations loaded with rifampicin and their respective controls. In conclusion, the encapsulation of rifampicin into a nanostructured delivery system not only provides a more stable drug formulation with increased shelf-life but also provides a safe dosage form for dermal application.

## 3. Materials and Methods

### 3.1. Materials

Rifampicin, rifampicin quinone European Pharmacopoeia Reference Standard, hyaluronic acid sodium salt (from Streptococcus equi), bovine serum albumin (BSA), penicillin–streptomycin solution (PenStrep^®^), and Hydranal^®^ (Sodium tartrate dihydrate) were purchased from Sigma-Aldrich Chemie GmbH (Steinheim/Schnelldorf, Germany). 3-Formylrifamycin was purchased from Cayman Chemical (Ann Arbor, MI, USA). The lipid Lipoid S 100 was kindly provided by Lipoid GmbH (Ludwigshafen, Germany). For hydrogel preparation, hydroxypropyl methylcellulose E4M Prem (4000 mPa·s) was purchased from Fagron GmbH & Co. KG (Glinde, Germany), and hydroxyethyl cellulose (10,000 mPa·s) was purchased from CAESAR & LORETZ GmbH (Hilden, Germany). The 3% collagen gel (from Chondrosia reniformis nardo) was kindly provided by Klinipharm GmbH (Eschborn, Germany). Fetal bovine serum (FBS, Biowest, Nuaillé, France), L(+)-ascorbic acid, and the cellulose ester dialysis membrane Spectra/Por^®^ Biotech with a molecular weight cutoff of 300 kDa were purchased from VWR International GmbH (Darmstadt, Germany). For Karl Fischer titration, the solvent Roti^®^ Hydroquant S and the titrant Roti^®^ Hydroquant T2 were purchased from Carl Roth GmbH (Karlsruhe, Germany). All organic solvents used for high-performance liquid chromatography analysis were gradient grade for liquid chromatography. Through all experiments, ultrapure water obtained from an Ultra Clear^®^ GP water purification system (Evoqua Water Technologies GmbH, Günzburg, Germany) was used. The Dispersion Releaser was manufactured from polyether ether ketone at the facilities of Goethe University (Frankfurt/Main, Germany).

### 3.2. Preparation of the Liposomes, Incorporation into Hydrogels, and Freeze-Drying

Rifampicin-loaded liposomes were manufactured using the thin lipid film hydration method [42]. A more detailed description of the preparation of the liposomes was reported previously [15]. Briefly, Lipoid S100 (≥94% of phosphatidylcholine) and rifampicin were dissolved in methanol which was then evaporated using a rotary evaporator (Büchi Rotavapor R-114, Büchi Labortechnik AG, Flawil, Schweiz). Afterward, the resulting lipid film was hydrated with phosphate-buffered saline (PBS) at a pH of 7.4 supplemented with 0.2 mg/mL L(+)-ascorbic acid to inhibit the oxidation of the drug. The size of the multilamellar vesicles (MLV) was adjusted in 11 extrusion cycles through a 400 nm polycarbonate membrane and 21 extrusion cycles through a 100 nm membrane with the help of a LiposoFast-Basic Extruder from Avestin, Inc. (Ottawa, Canada). A centrifugation step (10,000 rpm, 15 min) was carried out to remove the nonencapsulated fraction of rifampicin (Centrifuge 5430 R with rotor FA-45-30-11, Eppendorf AG, Hamburg, Germany). The vesicles were encapsulated into two different hydrogel formulations. Before the final formulation prototypes were selected, several excipients were evaluated.

#### 3.2.1. Nanostructured Hydrogels Based on Cellulose

The cellulose derivatives hydroxypropyl methylcellulose (HPMC), methylcellulose (MC), and hydroxyethyl cellulose (HEC) were tested at different concentrations to create a stable hydrogel formulation. Sucrose was added as a lyoprotectant in all preparations to enable subsequent freeze-drying. The gelling agents were added in small aliquots under mild agitation to the rifampicin liposomes followed by a swelling time of 1 h. A summary of the evaluated compositions is presented in Table 3.

After hydrogel preparation and characterization, the formulations were freeze-dried over 2 days using a Christ Epsilon 2–4 LSC freeze-dryer (Martin Christ Gefriertrocknungsanlagen GmbH, Osterode am Harz, Germany). A summary of the process parameters is provided in Table S1 (Supplementary Materials).

Several quality attributes were determined before and after the freeze-drying process. The aqueous dispersions were tested for their particle size, size distribution, zeta potential, and drug content, as explained in more detail in the later sections. After freeze-drying, the visual appearance of the freeze-dried cake, the redispersion time, and the residual moisture were evaluated. To quantify the water content, a V20 volumetric Karl Fischer titrator (Mettler Toledo GmbH, Gießen, Germany) was used. The device was equipped with a 5 mL burette containing the titrant Roti^®^ Hydroquant T2 (2 mg/mL) and a two-component solvent Roti^®^ Hydroquant S.

#### 3.2.2. Nanostructured Hydrogel Based on Collagen and Hyaluronic Acid

For the preparation of the liposomal collagen–hyaluronic acid hydrogel, various concentrations of gelation agents and several lyoprotectants and cryoprotectants were added. The compositions are summarized in Table 1 (Section 2.2). After the addition of these excipients to the liposomes, the gel was allowed to hydrate for 1 h before further characterization or processing. The characterization steps involved the determination of the particle size, size distribution, surface charge of the colloids, and drug content. The characterization steps are described in more detail in the later sections. A freeze-drying protocol was developed based on experiences with previous formulations. Sucrose and trehalose were identified as the most suitable lyoprotectants. In addition, freeze–thaw studies were conducted to identify suitable cryoprotectants. For this purpose, rifampicin-loaded liposomes were added to an aqueous solution comprising 0.5% collagen gel and various cryoprotectants. The samples were frozen at −80 °C (CryoCube F570H, Eppendorf SE, Hamburg, Deutschland) for 2.5 h and then allowed to thaw at room temperature. The cryoprotectants included dimethyl sulfoxide (DMSO) (0.5 M and 1 M), glycerol (0.5 M and 1 M), L-arginine (100 nM, 200 nM, and 400 nM), and PEG 400 (5% and 10%). For the freeze-dried samples, the visual appearance of the freeze-dried cake, the redispersion time, and residual moisture were also determined. The final process design is presented in Figure 3D (Section 2.2) and Table S2 (Supplementary Materials). The residual water content of the lyophilizates was determined using a V20 volumetric Karl Fischer titrator (Mettler Toledo GmbH, Gießen, Germany) as described for the HMPC gel (Section 3.2.1).

### 3.3. Quantification of Drug Content

The nanostructured hydrogels were analyzed for their rifampicin content, as well as for the presence of their main degradation products rifampicin quinone (RQ) and 3-formylrifamycin (3-FR). For the quantification, an HPLC system (Chromaster, VWR Hitachi, Tokyo, Japan) equipped with a photodiode array detector (no. 5430), a column oven (no. 5310), an autosampler (no. 5260), and a pump (no. 5160) was used. All samples were diluted with a mobile phase composed of 70% [*v*/*v*] acetonitrile and 30% [*v*/*v*] of a 10 mM dibasic sodium hydrogen phosphate buffer of a pH 6.8 and homogenized for 30 min in a thermomixer (Thermomixer comfort, Eppendorf AG, Hamburg, Germany) at 750 revolutions per minute (rpm) and 25 °C. Afterward, the samples were centrifuged at 10,000 rpm and 25 °C for 15 min (Centrifuge 5430 R with rotor FA-45-30-11, Eppendorf AG, Hamburg, Germany), and supernatants were injected into HPLC. The stationary phase consisted of a reversed-phase column (Zorbax Eclipse XDB-C18, 250 × 4.6 mm, pore size 80 Å, particle size 5 µm, Agilent Technologies Deutschland GmbH, Waldbronn, Germany) and a pre-column of the same material, which were kept at a constant temperature of 30 °C. A gradient program with a flow rate of 1.0 mL/min was used for the separation. In the first 2 min of the run time, the mobile phase comprised 38% [*v*/*v*] acetonitrile and 62% [*v*/*v*] of a 10 mM dibasic sodium hydrogen phosphate buffer at a pH of 6.8. After that, the acetonitrile fraction was increased to 70% [*v*/*v*] for 9 min. Finally, the initial composition of the mobile phase was reinjected over 3 min and maintained for another 5 min. All compounds were detected at a wavelength of 254 nm.

For rifampicin, linearity was determined over a concentration range of 0.1 µg/mL to 100 µg/mL, and the limit of detection (LOD) and limit of quantification (LOQ) were determined to be 0.05 µg/mL and 0.1 µg/mL, respectively. For RQ and 3-FR, linearity was confirmed over the concentration range of 0.05 to 100 µg/mL, and the LODs and LOQs were determined to be 0.02 µg/mL and 0.05 µg/mL, respectively.

### 3.4. Characterization of Nanostructured Hydrogels by DLS

The hydrogels were characterized for size (z-average), polydispersity index (PDI), and zeta potential of the incorporated liposomes using dynamic light scattering (DLS). The measurements were performed with a Malvern Zetasizer Nano-ZS (Malvern Panalytical Ltd., Malvern, UK) with a backscatter detector at an angle of 173°. The zeta potential was determined by using a Malvern dip cell. For all measurements, the gels were diluted 400-fold with PBS at a pH of 7.4, and all measurements were between six and nine attenuations.

### 3.5. Visualization of Nanostructured Hydrogels by TEM and Cryo-TEM

To complement the DLS measurements, further, the preparations were analyzed using cryogenic transmission electron microscopy (cryo-TEM). The system was equipped with an autoloader (Glacios, ThermoFisher Scientific, Waltham, MA, USA) to visualize the morphology and size of the liposomal formulations. Therefore, 3 µL of the formulations were added to a glow-discharged (3 s, Solarus Gatan) Quantifoil (2/1, copper) specimen support grid, blotted in a humidified atmosphere for 4 s using a Vitrobot (ThermoFisher Scientific, Waltham, MA, USA) and then plunged into liquid ethane. The autogrids were kept under liquid nitrogen, and the samples were investigated with an electron microscope at 200 kV. A Falcon 3 camera operated in linear mode served for taking the pictures at a pixel size of 2.5 Å (HPMC gel) or 1.35 Å (collagen–hyaluronic acid gel), respectively. At an underfocus of approximately 1.5 µm, movies with a total dose of around 20 e/Å^2^ were recorded and corrected for beam-induced motion (Motioncorr2 in Relion).

To investigate the hydrogels in the solid state, conventional transmission electron microscopy (TEM) was performed. The carbon-stabilized Formvar^®^ grids 200 mesh (Agar Scientific Ltd., Essex, UK) were subjected to glow discharge (5 mA, 20 s). The samples were diluted with double-distilled water (100-fold and 500-fold). Afterward, a volume of 10 uL of each sample was placed onto the grid. After 2 min, the remaining liquid was removed from the edge of the grid using filter paper. The grid was then stained with 1% gadolinium acetate for 45 s and blotted off with filter paper. Imaging was conducted in an FEI TECNAI SPIRIT G2 TEM (Field Electron and Ion Company, Hillsboro, OR, USA) operated at 100 KeV.

### 3.6. Stability Study over 6 Months

To compare the physical and chemical stability of the two hydrogels over time, a stability study following ICH guideline Q1A was performed [43]. For this purpose, both formulations (the lyophilizate and the hydrogel) were stored in a refrigerator at 5 °C ± 3 °C. In the case of the nanostructured collagen–hyaluronic acid gel, only the stability of the gel was investigated. After 0, 1, 3, and 6 months, the freeze-dried formulations were reconstituted with a predefined amount of ultrapure water and together with the gel samples examined for particle size, PDI, zeta potential (see Section 3.4), and drug content (see Section 3.3) of the liposomal carriers.

### 3.7. In Vitro Release Studies Using the Dispersion Releaser Technology

The release studies (n = 3) were performed using a USP apparatus 2 equipped with mini-vessels and the dispersion releaser (DR). This technology uses a dialysis-based setup, which was described in more detail previously [34,35,44]. Before the experiments, cellulose ester membranes with a molecular weight cutoff (MWCO) of 300 kDa were treated according to the manufacturer’s instructions and then mounted on the donor compartment of the DR. An MWCO of 300 kDa was used to ensure the separation of the rifampicin-loaded liposomes in the donor chamber from the free drug in the acceptor chamber. Earlier, we developed a new simulated wound fluid (SWF), which was used as the release medium for all experiments. SWF reflects the physiological conditions of wounds in terms of pH, buffer capacity, and protein content [15]. In addition, 1% [*v*/*v*] of a PenStrep^®^ solution was added to avoid microbial contamination, and media were deaerated by heating them to approximately 41 °C and stirring gently under a vacuum for 30 min (USP conditions). After that, 136 g of the release medium was filled in the acceptor compartment, and the temperature was maintained at 32 ± 0.5 °C. Both freeze-dried hydrogel formulations and the freeze-dried liposomal dispersion were reconstituted with a predefined amount of ultrapure water 1 h before the release experiments. Approximately 1.1 mg of the HPMC gel or 1.5 mg of the HA/collagen gel was dispensed into the donor chamber. A volume of 2.5 mL of the release medium was added. In the case of liposomal dispersion, approximately 1 mL was injected into the donor compartment using a syringe with a blunt needle (Sterican^®^ MIX, 1.2 × 40 mm, B.Braun, Melsungen, Germany). These amounts corresponded to an addition of 3.9 mg of rifampicin per vessel. During the release experiments, the stirring rate was set to 50 rpm. After 1, 2, 3, 4, 6, 8, 10, 24, and 48 h, samples of 500 µL were collected and replaced with fresh SWF. Before analyzing the drug content using HPLC the samples were treated as outlined in Section 2.3, except that the homogenization and centrifugation steps were performed at 4 °C.

### 3.8. Normalization of Drug Release Profiles

The drug permeation coefficient (k_M_) was determined to reduce the influence of membrane diffusion on the release profiles as described previously [34,36,37]. The respective models for the calculation of membrane permeation (https://exchange.iseesystems.com/public/matthiaswacker/dimec, accessed on 9 December 2022) and profile normalization (https://exchange.iseesystems.com/public/matthiaswacker/ptdr-reno, accessed on 9 December 2022) are available under a creative commons license. For this purpose, permeation experiments (n = 6) of the dissolved drug (3.9 mg∙mL^−1^) in wound fluid buffer (equivalent to SWF without proteins) were conducted at a pH of 7.4 using a cellulose ester membrane with an MWCO of 300 kDa. A nonlinear regression analysis of the permeation data was performed using the spreadsheet provided by Juhász et al. [45]. An acceptable fit was achieved using a first-order kinetic model. Accordingly, k_M_ describes the permeation process well enough for normalization [37]:

(1) $$\frac{dC_{a}}{dt}=\left[\frac{k_{m}\cdot A}{\delta \cdot V_{a}}\right]\times \left[C_{d}\left(t\right)-C_{a}\left(t\right)\right],$$

where C_a_ and C_d_ denote the concentrations in the acceptor and donor compartment, V_a_ represents the volume of the acceptor compartment, and A and δ denote the surface area and thickness of the membrane. The initial amount of rifampicin injected into the DR (Q_0_) and the volume of the donor compartment (V_d_) can be used to calculate the concentration of rifampicin in the donor compartment over time:

(2) $$C_{d}\left(t\right)=\frac{\left[Q_{0}-C_{a}\left(t\right)\cdot V_{a}\right]}{V_{d}}.$$

Equations (1) and (2) were combined as follows:

(3) $$\frac{dC_{a}}{dt}=\left[\frac{k_{m}\cdot A}{\delta \cdot V_{a}}\right]\cdot \left[\frac{Q_{0}-C_{a}\left(t\right)\cdot V_{a}\left(t\right)}{V_{d}}-C_{a}\left(t\right)\right].$$

Equation (3) was solved as follows:

(4) $$C_{a}\left(t\right)=\left[\frac{Q_{0}}{V_{a}+V_{d}}\right]\cdot \left\{1-\exp\left[\frac{A\cdot k_{M}\cdot \left(V_{a}\left(t\right)+V_{d}\right)\cdot t}{\delta \cdot V_{a}\left(t\right)\cdot V_{d}}\right]\right\}.$$

After simplifying Equation (4), the concentration of rifampicin in the acceptor compartment was defined as

(5) $$C_{a}\left(t\right)=C_{\infty }\cdot \left[1-\exp\left(k_{T}\cdot t\right)\right],$$

where C∞ denotes the rifampicin concentration when equilibrium is reached between the donor and acceptor compartments, and k_T_ represents the total diffusion coefficient. k_T_ is calculated every three seconds by combining measured time points with linear interpolation.

For further calculations, Equation (5) was solved as shown below.

(6) $$k_{T}=\frac{\ln\left(1-\frac{C_{a}\left(t\right)}{C_{\infty }}\right)}{t}=\left[\frac{A\cdot k_{M}\cdot \left(V_{a}\left(t\right)+V_{d}\right)}{\delta \cdot V_{a}\cdot V_{d}}\right],$$

such that the k_M_ value could be determined for each k_T_ value:

(7) $$k_{M}=\left[\frac{k_{T}\cdot \delta \cdot V_{a}\cdot V_{d}}{A\cdot \left(V_{a}\left(t\right)+V_{d}\right)}\right].$$

In addition, the model takes into account that deviations from the assumed first-order permeation are more likely to occur during the early sampling time and in the plateau phase, for which reason an average k_M_ was calculated from k_M_ values in a range between 15% and 85% permeation, as presented below.

(8) $$k_{M-Average}=\frac{1}{n_{15\%-85\%}}\cdot \overset{15\%}{\underset{85\%}{\sum }}k_{M}.$$

In this range, a constant flux between donor and acceptor compartments was reached. An average k_M_ was calculated accordingly:

(9) $$C_{d}\left(t\right)=\left(\frac{\Delta C_{a}}{\Delta dt}\right)\cdot \left[\frac{\delta \cdot V_{a}}{k_{M}\cdot A}\right]+C_{a}\left(t\right).$$

Lastly, the total amount of released rifampicin from the liposomal formulations (Q_r_) was defined as

(10) $$Q_{r}\left(t\right)=C_{d}\left(t\right)\cdot V_{d}+C_{a}\left(t\right)\cdot V_{a}.$$

### 3.9. Rheological Characterization of the HPMC Gel

The rheological behavior of the HPMC gel was examined by using the Modular Compact Rheometer MCR 302 equipped with a plate-Peltier temperature control device. The flow behavior of the liposomal gel was investigated before and after freeze-drying and at two different temperatures: 25 °C and 32 °C (to mimic the temperature of the skin surface). All samples were examined in triplicate. Before starting the measurements, the system was calibrated with a viscosity and density reference standard 26 cP (Paragon Scientific Limited, Prenton, UK) and a 2% HPMC gel (according to the certificate of analysis: 3354 mPa·s). To measure the flow behavior a cone-and-plate system as described in the USP guideline (method III, chapter 912) and a method suggested in the Application Report “Viscosity Measurements of Methylcellulose Solutions Used for Pharmaceutical Products” by Anton Paar was used [46,47]. For the measurements, approximately 700 µL of the formulations were placed on top of the plate with a diameter of 50 mm and allowed to reach thermal equilibrium. In the next step, the shear rate was continuously increased from 0.01–1000 1/s.

### 3.10. In Vitro Skin Irritation of Rifampicin Liposomes and HPMC Gel

To assess the biocompatibility of the developed liposomal HPMC gel, the liposomal dispersion and gel were investigated for skin viability using the MTT (3-(4,5-dimethylthiazol-2-yl)-2,5-diphenyltetrazolium bromide) assay. The experiments were conducted according to the protocol described in the Organization for Economic Co-operation and Development (OECD) test guideline 439 [41]. Before the experiments, the freeze-dried formulations were reconstituted with a predefined volume of ultrapure water and allowed to hydrate for 1 h before use. Next, 25 µL of the dispersion or gel formulations were applied to the surface of the reconstructed human epidermis (RHE) generated by a protocol described by Groeber et al. [48]. After the respective treatment time (35 min), the RHE was carefully rinsed eight times with 600 µL of PBS each and placed in 200 µL of MTT solution (1 mg/mL, Sigma-Aldrich Chemie GmbH, Steinheim, Germany) for 3 h in a humidified atmosphere at 37 °C and 5% CO2. After that, the MTT reduction was quantified by extracting the precipitated blue formazan salt with 2 mL of 2-propanol and, thus, measuring the optical density of the extract at 570 nm using a plate reader (Infinite^®^ 200 PRO M Nano, Tecan Group Ltd., Männedorf, Switzerland). The relative tissue viability was calculated by correcting the data using 2-propanol as blank and then normalizing the corrected optical density values to the negative control which was set to 100%.

### 3.11. Statistical and Graphical Analysis

All data are indicated as the mean value ± standard deviation (SD) using Microsoft Excel (Microsoft, Redmond, WA, USA) for the calculation. All experiments were conducted in triplicate (n = 3), except the investigations of the in vitro skin irritation which were conducted in duplicate (n = 2). For the calculation of the drug permeation coefficient (kM), as well as for the normalization of the cumulative release profiles, Stella^®^ Architect (V2.1.5, isee systems, Lebanon, PA, USA) was used. The f2 values were also determined using Microsoft Excel (Microsoft, Redmond, WA, USA). The characterization profiles (Figure 1, Figure 2 and Figure 4), release profiles, and viscosity profiles of HPMC gel were illustrated using OriginPro 2022 (OriginLab Corporation, Northhampton, MA, USA).

## 4. Conclusions

The present investigation was aimed at the development of a stable formulation prototype of rifampicin for further characterization in vitro and in vivo. Oxidation and hydrolysis of the compound in presence of water pose a major challenge. Accordingly, the liposomal dispersion was embedded into a hydrogel network and freeze-dried. Two gelation agents including HPMC, and a combination of hyaluronic acid and collagen led to the formation of a stable hydrogel. Marine collagen is an interesting excipient that could potentially lead to synergistic pharmacological effects. After freeze-drying, both hydrogels were redispersed within an acceptable time and contained low levels of residual water. Furthermore, the visual appearance of the freeze-dried cakes was acceptable. However, morphological characterization of the collagen–hyaluronic acid gel revealed an increase in the vesicle size, potentially due to interactions between phosphatidylcholine and collagen. Additionally, a faster release of rifampicin indicated a reduced encapsulation of the drug. Hence, the HPMC gel was further developed. Freeze-drying led to an easy-to-use formulation with optimal stability characteristics and a highly reproducible manufacturing process.

## Figures and Tables

**Table 1:** Summary of the formulations evaluated using various hyaluronic acid and collagen concentrations, as well as different types of cryo-/lyoprotectants.

	No.	Hyaluronic Acid Concentration [%]	Collagen Gel Concentration [%]	Cryoprotection/Lyoprotection	Cryoprotectant Concentration [%]	Cake Appearance after Freeze-Drying	Size Measurements (DLS)	Freeze-Drying Process
**A**	1	0.5	1.0	Trehalose	20	Collapsed cake	-	Table S1
	2	0.5	1.0	Sucrose	10	Collapsed cake	-	
	3	0.5	1.0	Sucrose	20	Collapsed cake	-	
	4	0.5	0.5	Trehalose	20	Collapsed cake	-	
	5	0.5	0.5	Sucrose	10	Collapsed cake	-	
	6	0.5	0.5	Sucrose	20	Collapsed cake	-	
	7	1.0	1.0	Sucrose	20	Collapsed cake	-	
	8	1.0	0.5	Sucrose	20	Collapsed cake	-	
**B**	1	0.5	0.5	Sucrose	10	Melted cake	>2 µm	Figure 3C
	2	0.5	1.0	Sucrose	10	Melted cake	>2 µm	
	3	0.5	1.5	Sucrose	10	Melted cake	-	
	4	0.5	0.5	Sucrose/PEG 4000	10/5	Melted cake	>2 µm	
	5	0.5	0.5	Sucrose/dextran	10/5	Melted cake	>6 µm *	
	6	0.5	0.5	Sucrose	20	Melted cake	>2 µm	
	7	0.5	0.5	Sucrose/mannitol	10/5	Melted cake	>2 µm	
**C**	1	0.5	0.5	Sucrose	10	Well-defined cake *	>2 µm	Figure 3D Table S2
	2	0.5	1.0	Sucrose	10	Melted cake	>2 µm	
	3	0.4	0.5	Sucrose	20	Well-defined cake	>6 µm *	
	4	0.5	0.5	Sucrose/PEG 4000	10/5	Well-defined cake *	>6 µm *	
	5	0.5	0.5	Sucrose/dextran	10/5	Well-defined cake *	>6 µm *	
	6	0.5	0.5	Sucrose/mannitol	10/5	Well-defined cake	- **	
	7	-	0.5	Sucrose/DMSO	10/0.5 M	Melted cake	>2 µm	
	8	-	0.5	Sucrose/DMSO	10/1 M	Collapsed cake	-	
	9	-	0.5	Sucrose/PEG 400	10/5	Melted cake	>2 µm	
	10	-	0.5	Sucrose/PEG 400	10/10	Melted cake	>6 µm *	
	11	0.5	0.5	Sucrose/DMSO	10/0.5 M	Melted cake	>2 µm	
	12	0.5	0.5	Sucrose/DMSO	10/1 M	Collapsed cake	-	
	13	0.5	0.5	Sucrose/PEG 400	10/10	Well-defined cake	>2 µm	
	14	0.5	0.5	Sucrose/PEG 400	10/15	Collapsed cake	-	

* The z-average is larger than the upper size limit. ** The sample could not be completely redispersed.

**Table 2:** DLS data of the hydrogel preparations.

	HPMC Gel		Collagen–Hyaluronic Acid Gel	
Characteristic	Before FD	After FD	Before FD	After FD
**Particle size [nm]**	103 ± 2	116 ± 1	113 ± 1	2285 ± 325 410 ± 134 *
**PDI**	0.117 ± 0.012	0.181 ± 0.007	0.099 ± 0.012	0.64 ± 0.203
**Zeta potential [mV]**	−3.29 ± 0.464	−2.67 ± 0.374	−9.37 ± 1.23	−4.35 ± 0.77

* The particle size was determined from the cryo-TEM images counting a total of 100 particles. FD, freeze-drying.

**Table 3:** Summary formulation approaches using different celluloses and various sucrose concentrations.

	Formulation	Cellulose	Cellulose Concentration [%]	Sucrose Concentration [%]
**A**	1	HPMC	2.5	5
	2	HPMC	3.0	5
	3	MC	2.0	5
	4	MC	2.5	5
	5	HEC	2.0	5
	6	HEC	2.5	5
**B**	7	HPMC	1.0	5
	8	HPMC	1.0	10
	9	HPMC	2.0	5
	10	HPMC	2.0	10
	11	HEC	0.5	5
	12	HEC	0.5	10
	13	HEC	1.0	5
	14	HEC	1.0	10

HPMC, hydroxypropylmethylcellulose, 4000 mPa·s. MC, methylcellulose, 8000 mPa·s. HEC, hydroxyethylcellulose, 10,000 mPa·s.

## Data Availability

Data will be available on request.

## Supplementary Materials

The following supporting information can be downloaded at https://www.mdpi.com/article/10.3390/ijms232416207/s1.

## References

[1] Sen C.K., Gordillo G.M., Roy S., Kirsner R., Lambert L., Hunt T.K., Gottrup F., Gurtner G.C., Longaker M.T. (2009). Human skin wounds: A major and snowballing threat to public health and the economy. Wound Repair Regen..

[2] Ashtikar M., Wacker M.G. (2018). Nanopharmaceuticals for wound healing—Lost in translation?. Adv. Drug Deliv. Rev..

[3] Iacob A.T., Lupascu F.G., Apotrosoaei M., Vasincu I.M., Tauser R.G., Lupascu D., Giusca S.E., Caruntu I.-D., Profire L. (2021). Recent Biomedical Approaches for Chitosan Based Materials as Drug Delivery Nanocarriers. Pharmaceutics.

[4] Iacob A.-T., Drăgan M., Ionescu O.-M., Profire L., Ficai A., Andronescu E., Confederat L.G., Lupașcu D. (2020). An Overview of Biopolymeric Electrospun Nanofibers Based on Polysaccharides for Wound Healing Management. Pharmaceutics.

[5] Acuña L., Hamadat S., Corbalán N.S., González-Lizárraga F., Dos-Santos-Pereira M., Rocca J., Díaz J.S., Del-Bel E., Papy-García D., Chehín R.N. (2019). Rifampicin and Its Derivative Rifampicin Quinone Reduce Microglial Inflammatory Responses and Neurodegeneration Induced In Vitro by α-Synuclein Fibrillary Aggregates. Cells.

[6] Bi W., Zhu L., Wang C., Liang Y., Liu J., Shi Q., Tao E. (2011). Rifampicin inhibits microglial inflammation and improves neuron survival against inflammation. Brain Res..

[7] Wang X., Grace P.M., Pham M.N., Cheng K., Strand K.A., Smith C., Li J., Watkins L.R., Yin H. (2013). Rifampin inhibits Toll-like receptor 4 signaling by targeting myeloid differentiation protein 2 and attenuates neuropathic pain. FASEB J..

[8] Haferland I., Wallenwein C.M., Ickelsheimer T., Diehl S., Wacker M.G., Schiffmann S., Buerger C., Kaufmann R., Koenig A., Pinter A. (2022). Mechanism of anti-inflammatory effects of rifampicin in an ex vivo culture system of hidradenitis suppurativa. Exp. Dermatol..

[9] Gibson D.J., Schultz G.S. (2013). Molecular Wound Assessments: Matrix Metalloproteinases. Adv. Wound Care.

[10] Sharma A., Puri V., Kumar P., Singh I. (2020). Rifampicin-Loaded Alginate-Gelatin Fibers Incorporated within Transdermal Films as a Fiber-in-Film System for Wound Healing Applications. Membranes.

[11] Gurel M.S., Naycı S., Turgut A.V., Bozkurt E.R. (2015). Comparison of the effects of topical fusidic acid and rifamycin on wound healing in rats. Int. Wound J..

[12] Li J., Zhu M., Rajamani S., Uversky V.N., Fink A.L. (2004). Rifampicin inhibits alpha-synuclein fibrillation and disaggregates fibrils. Chem. Biol..

[13] Jindal K., Chaudhary R.S., Singla A.K., Gangwal S., Khanna S. (1995). Effect of buffers and pH on Rifampicin stability. Die Pharm. Ind..

[14] Prankerd R.J., Walters J.M., Parnes J.H. (1992). Kinetics for degradation of rifampicin, an azomethine-containing drug which exhibits reversible hydrolysis in acidic solutions. Int. J. Pharm..

[15] Wallenwein C.M., Ashtikar M., Hofhaus G., Haferland I., Thurn M., König A., Pinter A., Dressman J., Wacker M.G. (2022). How Wound Environments Trigger the Release from Rifampicin-loaded Liposomes. Int. J. Pharm..

[16] Dias F., Duarte C., van de Ven T., Godbout L. (2013). Cellulose and Its Derivatives Use in the Pharmaceutical Compounding Practice. Cellulose—Medical, Pharmaceutical and Electronic Applications.

[17] Kamel S., Ali N., Jahangir K., Shah S.M., El-Gendy A.A. (2008). Pharmaceutical significance of cellulose: A review. Express Polym. Lett..

[18] Swatschek D., Schatton W., Kellermann J., Müller W.E., Kreuter J. (2002). Marine sponge collagen: Isolation, characterization and effects on the skin parameters surface-pH, moisture and sebum. Eur. J. Pharm. Biopharm..

[19] Friess W. (1998). Collagen—Biomaterial for drug delivery1Dedicated to Professor Dr. Eberhard Nürnberg, Friedrich-Alexander-Universität Erlangen-Nürnberg, on the occasion of his 70th birthday. Eur. J. Pharm. Biopharm..

[20] Takeda U., Odaki M., Yokota M., Sasaki H., Niizato T., Kawaoto H., Watanabe H., Ito T., Ishiwatari N., Hayasaka H. (1982). Acute and subacute toxicity studies on collagen wound dressing (CAS) in mice and rats. J. Toxicol. Sci..

[21] Chattopadhyay S., Raines R.T. (2014). Review collagen-based biomaterials for wound healing. Biopolymers.

[22] Fleck C.A., Chakravarthy D. (2007). Understanding the mechanisms of collagen dressings. Adv. Skin Wound Care.

[23] Aramwit P., Ågren M.S. (2016). Introduction to biomaterials for wound healing. Wound Healing Biomaterials.

[24] Hussain Z., Thu H.E., Katas H., Bukhari S.N.A. (2017). Hyaluronic Acid-Based Biomaterials: A Versatile and Smart Approach to Tissue Regeneration and Treating Traumatic, Surgical, and Chronic Wounds. Polym. Rev..

[25] Sze J.H., Brownlie J.C., Love C.A. (2016). Biotechnological production of hyaluronic acid: A mini review. 3 Biotech.

[26] Salwowska N.M., Bebenek K.A., Żądło D.A., Wcisło-Dziadecka D.L. (2016). Physiochemical properties and application of hyaluronic acid: A systematic review. J. Cosmet. Dermatol..

[27] Heinemann S., Ehrlich H., Douglas T., Heinemann C., Worch H., Schatton W., Hanke T. (2007). Ultrastructural studies on the collagen of the marine sponge Chondrosia reniformis Nardo. Biomacromolecules.

[28] Ozcelikkale A., Han B. (2016). Thermal Destabilization of Collagen Matrix Hierarchical Structure by Freeze/Thaw. PLoS ONE.

[29] Kodama T., Takeuchi M., Wakiyama N., Terada K. (2014). Optimization of secondary drying condition for desired residual water content in a lyophilized product using a novel simulation program for pharmaceutical lyophilization. Int. J. Pharm..

[30] Villa Nova M., Janas C., Schmidt M., Ulshoefer T., Gräfe S., Schiffmann S., de Bruin N., Wiehe A., Albrecht V., Parnham M.J. (2015). Nanocarriers for photodynamic therapy-rational formulation design and medium-scale manufacture. Int. J. Pharm..

[31] Bai K., Barnett G.V., Kar S.R., Das T.K. (2017). Interference from Proteins and Surfactants on Particle Size Distributions Measured by Nanoparticle Tracking Analysis (NTA). Pharm. Res..

[32] Marques M.R.C., Choo Q., Ashtikar M., Rocha T.C., Bremer-Hoffmann S., Wacker M.G. (2019). Nanomedicines—Tiny particles and big challenges. Adv. Drug Deliv. Rev..

[33] Mast M.-P., Modh H., Champanhac C., Wang J.-W., Storm G., Krämer J., Mailänder V., Pastorin G., Wacker M.G. (2021). Nanomedicine at the crossroads—A quick guide for IVIVC. Adv. Drug Deliv. Rev..

[34] Janas C., Mast M.P., Kirsamer L., Angioni C., Gao F., Mantele W., Dressman J., Wacker M.G. (2017). The dispersion releaser technology is an effective method for testing drug release from nanosized drug carriers. Eur. J. Pharm. Biopharm..

[35] Wallenwein C.M., Nova M.V., Janas C., Jablonka L., Gao G.F., Thurn M., Albrecht V., Wiehe A., Wacker M.G. (2019). A dialysis-based in vitro drug release assay to study dynamics of the drug-protein transfer of temoporfin liposomes. Eur. J. Pharm. Biopharm..

[36] Mast M.-P., Modh H., Knoll J., Fecioru E., Wacker M.G. (2021). An Update to Dialysis-Based Drug Release Testing-Data Analysis and Validation Using the Pharma Test Dispersion Releaser. Pharmaceutics.

[37] Xie L., Beyer S., Vogel V., Wacker M.G., Mantele W. (2015). Assessing the drug release from nanoparticles: Overcoming the shortcomings of dialysis by using novel optical techniques and a mathematical model. Int. J. Pharm..

[38] Batheja P., Sheihet L., Kohn J., Singer A.J., Michniak-Kohn B. (2011). Topical drug delivery by a polymeric nanosphere gel: Formulation optimization and in vitro and in vivo skin distribution studies. J. Control. Release.

[39] Binder L., Mazál J., Petz R., Klang V., Valenta C. (2019). The role of viscosity on skin penetration from cellulose ether-based hydrogels. Skin Res. Technol..

[40] Ferdous A.J. (1992). Viscosity and stability stijdies of hydroxypropyl methylcellulose polymer solutions. Pak. J. Pharm. Sci..

[41] OECD (2015). Test No. 439: In Vitro Skin Irritation: Reconstructed Human Epidermis Test Method.

[42] Bangham A.D., Standish M.M., Watkins J.C. (1965). Diffusion of univalent ions across the lamellae of swollen phospholipids. J. Mol. Biol..

[43] ICH Harmonised Tripartite Guideline Stability Testing of New Drug Substances and Products Q1A(R2). https://database.ich.org/sites/default/files/Q1A%28R2%29%20Guideline.pdf.

[44] Jablonka L., Ashtikar M., Gao G., Jung F., Thurn M., Preuß A., Scheglmann D., Albrecht V., Röder B., Wacker M.G. (2019). Advanced in silico modeling explains pharmacokinetics and biodistribution of temoporfin nanocrystals in humans. J. Control. Release.

[45] Juhász Á., Ungor D., Berta K., Seres L., Csapó E. (2021). Spreadsheet-based nonlinear analysis of in vitro release properties of a model drug from colloidal carriers. J. Mol. Liq..

[46] United States Pharmacopeia (2022). General Chapter,〈912〉 Viscosity—Rotational Methods.

[47] Paar A. Viscosity Measurements of Methylcellulose Solutions Used for Pharmaceutical Products. https://www.anton-paar.com/corp-en/services-support/document-finder/application-reports/viscosity-measurements-of-methylcellulose-solutions-used-for-pharmaceutical-products/.

[48] Groeber F., Schober L., Schmid F.F., Traube A., Kolbus-Hernandez S., Daton K., Hoffmann S., Petersohn D., Schäfer-Korting M., Walles H. (2016). Catch-up validation study of an in vitro skin irritation test method based on an open source reconstructed epidermis (phase II). Toxicol. Vitr..

