# Supplementary material for: Pharmaceutical Development of Nanostructured Vesicular Hydrogel Formulations of Rifampicin for Wound Healing
Source: Int J Mol Sci. 2022 Dec 19;23(24):16207. doi: 10.3390/ijms232416207 (PMC9788359; doi:10.3390/ijms232416207)
Supplement: Supplementary file 1 [file ijms-23-16207-s001.zip › ijms-2077824-supplementary.pdf]

Supplementary Materials to the article:

Pharmaceutical Development of Nanostructured Vesicular Hydrogel Formulations of Rifampicin for Wound Healing

Chantal M. Wallenwein [1], Verena Weigel [2], Götz Hofhaus [3], Namrata Dhakal [4], Wolfgang Schatton[5], Svetlana Gelperina [6], Florian K. Groeber-Becker [2], Jennifer Dressman [1] and Matthias G. Wacker [4,*]

[1] Fraunhofer Institute for Translational Medicine and Pharmacology ITMP, Theodor-Stern-Kai 7, 60596 Frankfurt am Main, Germany
[2] Translational Center for Regenerative Therapies, Fraunhofer Institute for Silicate Research ISC, Neunerplatz 2, 97082 Würzburg, Germany
[3] Cryo Electron Microscopy, CellNetworks, BioQuant, Universitätsklinikum Heidelberg, 69120 Heidelberg, Germany
[4] Department of Pharmacy, Faculty of Science, National University of Singapore, 4 Science Drive 2, Singapore 117544, Singapore
[5] Klinipharm GmbH, Hauptstraße 23, 65760 Eschborn, Germany
[6] Faculty of Chemical and Pharmaceutical Technologies and Biomedical Drugs, D. Mendeleev University of Chemical Technology of Russia, Miusskaya pl. 9, 125047 Moscow, Russia
* Correspondence: matthias.g.wacker@nus.edu.sg

Table S1: Process parameters during the freeze-drying process of liposomal HPMC gel.

Name	Duration [hh:mm]	Time [hh:mm]	Vacuum pressure [mbar]	Temperature [°C]
**Freezing**	01:00	01:00	1024	-60
**(Load at end of section)**				
**Freezing**	02:30	03:30	1024	-60
**Primary Drying**	01:00	04:30	0.94	-30
	05:00	09:30	0.006	-10
	31:00	40:30	0.006	-10
**Secondary Drying**	00:30	41:00	0.006	10
	10:00	51:00	0.006	20
	4:00	55:00	1.024	20

Table S2: Process parameters during the final freeze-drying process of liposomal hyaluronic acid-collagen gel.

Name	Duration [hh:mm]	Time [hh:mm]	Vacuum pressure [mbar]	Temperature [°C]
**Freezing**	01:00	01:00	1024	-60
**(Load at end of section)**				
**Freezing**	02:30	03:30	1024	-60
**Primary Drying**	01:00	04:30	0.94	-60
	02:00	06:30	0.94	-60
	01:00	07:30	0.52	-60
	03:00	10:30	0.52	-60
	01:00	11:30	0.20	-60
	05:00	16:30	0.20	-60
	01:00	17:30	0.07	-60
	10:00	27:30	0.07	-60
	30:00	57:30	0.07	-55
	20:00	77:30	0.07	-50
	20:00	97:30	0.07	-45
	20:00	117:30	0.07	-40
	20:00	137:30	0.07	-35
	20:00	157:30	0.07	-30
	20:00	177:30	0.07	-25
	20:00	197:30	0.07	-20
	20:00	207:30	0.07	-10
	10:00	217:30	0.07	-10
**Secondary Drying**	05:00	222:30	0.006	10
	10:00	232:30	0.006	20
	4:00	236:30	1.024	20
